# Comparison of efficacy of procalcitonin (PCT) and serum C-reactive protein (CRP) as biochemical markers of sepsis in pediatric intensive care unit (ICU)

**DOI:** 10.12669/pjms.42.6.15569

**Published:** 2026-06

**Authors:** Ali Abdur Rahman, Asfand Tariq, Lubna Riaz

**Affiliations:** 1 Ali Abdur Rahman, *MBBS*, Postgraduate Resident in Pediatric Medicine, Shaikh Zayed Hospital Lahore, Pakistan; 2 Asfand Tariq, *MBBS, FCPS (Pediatric Medicine)*, Assistant Professor, Department of Pediatric Medicine, Shaikh Zayed Hospital Lahore, Pakistan; 3 Lubna Riaz, *MBBS, MCPS (Pediatric Medicine), FCPS (Pediatric Medicine)*, Associate Professor, Department of Pediatric Medicine, Shaikh Zayed Hospital Lahore, Pakistan

**Keywords:** Biochemical markers, C-reactive protein, Procalcitonin, Pediatric intensive care unit, Sepsis

## Abstract

**Background & Objectives:**

Sepsis in pediatric population has a high mortality rate and global health impact. Explicit diagnostic testing is critical in order to ensure timely and accurate diagnosis. Our study is aimed at comparing the efficacy of CRP and PCT, keeping blood culture as gold standard, in diagnosing sepsis in pediatric population to improve treatment and survival.

**Methodology:**

This was a cross-sectional survey conducted at the Department of Pediatric Medicine at The Shaikh Zayed Hospital Lahore. Patients included were newly admitted neonates up to one month age with suspected sepsis, to the Pediatric Intensive Care Unit over a period of six months, i.e. January 3, 2025 to June 30, 2025. Data collection was done by interviewing parents using a structured proforma, CRP and PCT tests were done and their sensitivity, specificity, and diagnostic accuracy were calculated.

**Results:**

A total of 165 neonates were enrolled in this study. Male were 46.7% and female were 53.3%. The most common age of presentation was zero day of life (Range 0-23 day, Mean one day, median zero day). C-Reactive Protein had a sensitivity and specificity of 75.5% and 67.2% respectively; while, Procalcitonin had a significantly higher sensitivity and specificity 83.6% and 86.2% respectively. The most common culture growth organism was *Klebsiella* (44.4%); other organisms included *Pseudomonas* (26.7 %), *Acinetobacter* (20%), and *E.coli* (8.9%).

**Conclusions:**

Procalcitonin has a better sensitivity and specificity than C reactive protein in the early diagnosis of sepsis.

## INTRODUCTION

Diagnosis of sepsis remains challenging and new methods for recognizing inflammatory response and predicting mortality must be developed.^1^ Sepsis is a major cause of neonatal mortality, and rapid accurate diagnosis is often difficult due to its non-specific presentation. Improving the diagnostic testing will improve the outcomes in septic patients and reduce the antibiotic administration in aseptic patients. Newborn sepsis (NS) is a global health concern and a major cause of neonatal ICU admissions and leading cause of mortality in children. The PCT shows mild increase with severe viral infections, and inflammatory conditions of non-infectious origin. It is the most important diagnostic test for early neonatal sepsis, as it detects bacterial infection in two hours, rises rapidly in 2-4 hours, plateaus after 6-8 hours, and returns to normal after 24 hours.^2^ Due to its non-specific presentation, a timely and accurate diagnosis of NS is crucial. The global neonatal death rate due to sepsis is approximately 40%.^3^ Furthermore, in developing countries, lack of accurate assessment and limited medical resources lead to inaccurate diagnoses.^3^

In Pakistan, the lack of significant data and high neonatal mortality rates provide a rationale for assessing the impact of effective biomarkers like procalcitonin (PCT) in early diagnosis of sepsis.^4^ Although the gold standard diagnostic tool for sepsis is blood culture, it delays intervention due to prolonged waiting time, low positivity rate, and false negative results from low-colony count bacteremia in up to 60% of blood cultures.^4^,^5^ In contrast, CRP does not differentiate between bacterial and other infections, is more valuable in late-onset neonatal sepsis, and its levels may rise increasingly in other birth related events such as perinatal asphyxia, respiratory distress syndrome, brain hemorrhage, meconium aspiration syndrome and post-surgical period, thus misrepresents sepsis.^6^ The half-life of PCT is approximately 24 hours therefore levels drop with patients recovery.^7^ In comparison, CRP takes more than 12-24 hours to rise and remains elevated for up to three to seven days.^7^

The rationale for this study is limited utility, poor sensitivity and specificity of traditional biomarkers for bacterial infections, and the inability of gold standard diagnostic blood cultures to reflect the host immune response of inflammation or the onset of organ dysfunction, resulting in false positive or false negative reports. Since PCT increases earlier and normalizes more rapidly, it offers better disease detection and monitoring of its progression. Thus, a systematic use of PCT may have a positive effect on reducing antibiotic treatment, ICU stay, and better prediction of survival, making it a better option compared to CRP.

## METHODOLOGY

It was a cross-sectional study, via non-probability consecutive sampling using a proforma-based data collection. It included information regarding the age of patient, neonatal presenting complaints according to the mother and observed by the researcher, maternal complaints during pregnancy, and at the time of delivery. The results of PCT, CRP, and Blood culture status were taken from file record of patients. The study was conducted at the Department of Pediatric Medicine, at The Shaikh Zayed Hospital, Lahore, over a period of six months i.e. 3^rd^ January 2025 to 30^th^ June 2025. A Sample size of 165 cases was calculated using 95% confidence level, 90% power, with an expected prevalence of sepsis 29.8%, sensitivity and specificity of 69.0% and 77.0%, respectively, for CRP, for the diagnosis of neonatal sepsis with 13% margin of error.^4^

### Inclusion criteria:

- Newly admitted neonates up to one month age of both genders having any two to three signs (maternal fever, lethargy, febrile, feeding intolerance, cyanosis, bradycardia, tachycardia, tachypnea, seizures, hypotonia, apnea, respiratory distress, hepatomegaly, abdominal distension or vomiting) were included in the study.

### Exclusion criteria:

- Neonates coming with proven sepsis with ongoing treatment or with proven sepsis with congenital anomalies will not be considered.

### Ethical Approval:

It was approved from the hospital ethical committee (No. 02-TERC/NHRC/SZH/Int/SC/464 dated March 14, 2024),

### Data collection:

The data was collected while maintaining anonymity and confidentiality. After obtaining consent, the patients/parents were interviewed using a structured proforma regarding the patient’s age, clinical findings of neonate at presentation, and clinical findings of mother during pregnancy and at the time of delivery. Based on the results of blood culture, the study included two groups: one group of children with culture-proven sepsis and the other group with negative culture.

The demographic and clinical details of the patients were noted according to a structured proforma. Blood was collected from peripheral veins of the children with clinical signs/symptoms of sepsis before any interventions. Sample was collected for PCT, CRP measurements (1-2 ml), and blood culture (1-2 ml). PCT is based on immunochromatography and sample obtained via micro-pipette was analyzed. The cut off level > 1.1 ng/dl was considered positive for PCT. CRP sample was used to produce a 100 ul of dilated specimen and analyzed. The cut off level of > 10 mg/dl was considered positive.^4^ Suspected sepsis was defined as neonates with fever, hypothermia, tachycardia, tachypnea, bradypnea, cyanosis, apnea, lethargy or irritability, prematurity, vomiting or feeding difficulty, hypotonia, and seizures. Neonatal presentation, according to the history described by the mother and clinical findings observed by the researcher physician, was noted.

Sepsis was defined as CRP > 10 mg/dl, PCT > 1.1ng/l, and blood culture > 10^5^/HPF. The Gold standard test for marking sepsis is the blood culture sensitivity test. Blood culture positive was defined as detection of > 10^5^/HPF bacterial growth in a blood sample in an automated incubation system within five to six days. True positive were those with positive blood culture tests and positive marker of sepsis (i.e. CRP or PCT), and true negative were those with negative blood culture tests and negative marker of sepsis (i.e. CRP or PCT). Those with positive blood culture but negative marker of sepsis (i.e. CRP or PCT) were taken as false negative. Negative blood culture but positive marker of sepsis (i.e. CRP or PCT) were taken as false positive.

### Data analysis:

The categorical data like gender, clinical features, CRP findings, PCT findings, pathogen findings, and blood culture findings were measured as frequency and percentages. The two-by-two table was generated to present the following: true positive, true negative, false positive, and false negative. Based on these values, sensitivity, specificity, Positive predictive value (PPV), Negative predictive value (NPV), and diagnostic accuracy were calculated.

## RESULTS

The primary outcome of this study was comparison of the diagnostic accuracy of CRP and PCT in diagnosis of pediatric sepsis, keeping the blood culture as the gold standard. A total of 165 new patients presented over six months of this study, with a male-to-female ratio of 7:8 (77 males to 88 females). Our study focused primarily on neonatal age group. Most common age of presentation was within first 24 hours i.e. day zero ((Range 0-23 days) of life (i.e. soon after birth). The most common maternal risk factor was intrapartum fever; other risk factors included were UTI, PV leaking > 18 hours, and PROM > 18 hours. The most common presentation of neonate included feeding intolerance; other findings included tachypnea, lethargy, abdominal distension, bradypnea, and fits. These results are illustrated in Table-I.

**Table I:** Demographic characteristics.

Characteristic	N (%)
***Age***	
Up to 1 week	120 (72.7%)
1-2 week	30 (18.2%)
2week-1 month	15 (9.1%)
***Gender***	
Male	77 (46.7%)
Female	88 (53.3%)
***Maternal clinical findings***	
Intrapartum fever	58 (63.7%)
UTI	42 (46.2%)
PV leaking > 18 hours	20 (22%)
PROM > 18 hours	15 (16.5%)
***Neonate clinical findings***	
Reluctance to feed	120 (85.7%)
Tachypnea	115 (82.1%)
Lethargy	102 (72.9%)
Abdominal distension	85 (60.7%)
Bradypnea	29 (20.7%)
Fits	11 (7.9%)

The common diseases in our patients were early onset sepsis 116 (70%), late onset sepsis 28 (17%), congenital pneumonia 20 (12.1%), and meningitis 1 (0.6%). The most common culture growth organism was Klebsiella; other organisms included Pseudomonas, Acinetobacter, and E.coli. The results are illustrated in Table-II. The response rate to questioned patients was 100% and there were no reported refusals. The findings of CRP and PCT were correlated with blood culture results as shown in the Table-III. Sensitivity, specificity, and diagnostic accuracy of PCT was significantly higher than CRP shown in Table-IV.

**Table-II:** Blood culture organisms.

Organism	N (%)
Klebsiella	20 (44.4%)
Pseudomonas	12 (26.7%)
Acinetobacter	9 (20%)
E.coli	4 (8.9%)

**Table-III:** Comparison on CRP vs PCT with blood culture.

	Positive blood culture (49)	Negative blood culture (116)
***CRP***		
Positive	37	38
Negative	12	78
***PCT***		
Positive	41	16
Negative	8	100

**Table-IV:** Diagnostic parameters.

	CRP	PCT
Sensitivity	75.5%	83.6%
Specificity	67.2%	86.2%
Positive predictive value	49.3%	71.9%
Negative predictive value	86.7%	92.6%
Diagnostic accuracy	69.7%	85.4%

## DISCUSSION

In this study, the positive CRP test results were comparable between the patients with and without blood culture proven sepsis; however, the number of PCT positive patients with positive blood culture results was significantly higher than those with negative blood culture results. Furthermore, PCT was found to be more sensitive, more specific, and with better diagnostic accuracy compared to CRP. The most frequent organism observed in our study was Klebsiella followed by Pseudomonas, E.coli, and Acinetobacter. Although CRP is cost effective, in recent years, PCT has been considered as a specific biomarker for early diagnosis of neonatal sepsis.^8^,^9^

Our findings are comparable to other studies with Klebsiella being the most common organism followed by Pseudomonas and staphylococcus aureus. Kumar D et al. mentions sensitivity and specificity of CRP 80.6% and 44.2%, respectively, a trend similar to that seen in our study.^10^ Shen Y et al. shows higher values for both CRP and PCT; the sensitivity of 78.7% vs 93.8% and specificity 94.3% vs 91.8% respectively. Some studies show sensitivity and specificity for both CRP and PCT higher as compared to our results.^11^ Others show sensitivity and specificity of PCT was higher compared to CRP.^12^ Comparitive review show higher specificity for CRP and lower for PCT as compared to our results; PCT was observed as the more sensitive test and CRP as the more specific test.^13^ Similar results were shown in another study where CRP sensitivity 61% specificity 85%, and PCT sensitivity 80% and specificity 77% respectively.^14^ The study which show higher specificity for CRP compared to PCT could be due to variation in timing of sample collection, difference in study population, varied cut-off values, laboratory methods to measure CRP and PCT, and presence of other factors such as non-infective inflammatory conditions influencing the CRP levels thus increasing its specificity compared to PCT.^15^ Li JJ et al. show sensitivity and specificity of CRP as 87% and 33%, and PCT as 100% and 53% respectively.^16^ In comparison to our study, most studies show superiority of PCT over CRP in terms of sensitivity and specificity for diagnosis of sepsis.^17^ This was due to comparable diagnostic criteria, biomarker cut-off values, and timing of sample collection. The PCT level was observed in the lower range during infection or systemic inflammation, and higher levels are found during severe sepsis and septic shock, thus supporting the diagnosis of bacterial infection. 18

Pakistan being a community belonging to low socioeconomic class and an overburdened health care setting requires a rapid screening test for accurate diagnosis and timely management of neonatal sepsis. Thus, the current study findings have a beneficial outcome and is in accordance with our national, social, and demographic demands. Further large-scale studies are needed to assess, and support evidence of better diagnostic benefits of PCT. In conclusion, this study endorses the previously established precedence of better diagnostic accuracy of PCT than CRP, and its role in ruling out bacteremia in children with sepsis.^19^

Strengths: All the patients were newly admitted thus the data may closely represent sepsis criteria.

### Limitations:

Limited generalization to population due to collection from a single center. Our study was also for a short duration due to limitation of resources and man power.

## CONCLUSION

Rapid identification of infection has a major impact in the clinical course, management of disease, use of antibiotics, and the outcome of patients in an intensive care unit. PCT has shown to be significantly superior to CRP as a marker of clinical severity, and is more useful in early diagnosis of sepsis in neonatal patients under treatment at the pediatric intensive care units. Our study aims to highlight the areas in the healthcare system of Pakistan for considerable interventions to improve the outcome of children with sepsis.

### Author`s Contribution:

**AAR, AT and LR:** Conception and design of work, acquisition of data, analysis interpretation of data, drafting and critical revision of the Manuscript and final approval of version to be published. **AAR, AT and LR:** Critical Review, Accountable for all aspects of the work in ensuring that questions related to the accuracy or integrity of any part of the work are appropriately investigated and resolved.
